# Summary of Monthly Reports of Medical Department of Southern Illinois Penitentiary

**Published:** 1882-10

**Authors:** H. Z. Gill

**Affiliations:** Physician to Southern Penitentiary


### ARTICLE III.

SUMMARY OF MONTHLY REPORTS OF THE MEDICAL DEPARTMENT OF THE SOUTHERN ILLINOIS PENITENTIARY, CHESTER, ILL. For the year commencing August 1, 1881, and ending July 31, 1882. By H. Z. GILL, A.M., M.D., Physician to Southern Penitentiary.

Having no "idle house" connected with the institution, when men are not able for light duty, from sickness or debility, they are reported in hospital or in *sick cell;* and many are in hospital who would otherwise be in a convalescent ward or idle house, if there were such a place. Hence the report of days lost in hospital and in sick cell is much larger at times than it otherwise would be.

At the time of changing the location and building occupied as hospital (about May 20), the culinary department of the hospital was discontinued and the provisions supplied from the general officers' kitchen. About the beginning of the year, the number attending morning sick-call somewhat increased, as the number of convicts increased; and, also, there was more care in recording all who came to the call, whether they received any important treatment or not. About that time there were other but temporary causes for the increase. During the early months of the year there was a considerable amount of diarrhœa, dependent upon a combination of causes; partly from the diet, and partly from the water, the river at the time being very high. After commencing the use of cistern-water, and using less fresh meat and more vegetables, the difficulty diminished; but the disturbances of the bowels, with the necessarily routine diet, will always be a trouble to be met, especially in the class of men here found.

The cases of intermittent fever (single attacks numbering some hundreds, many of them not taken into hospital) were generally easily controlled with alkaline aperients, and the antiperiodic cinchonidia sulphate. The remittent, malarial and continued forms of fever were of moderate severity. The cases of typhoid fever, eight in number, were mostly well defined, and the majority quite severe. The three commencing in April were especially marked in the nervous element of the cases. They were quite delirious, two of them sleepless and noisy for several nights, but these two recovered, one of them after suffering a perforation of the membrana tympani. From this latter trouble, however, he ultimately entirely recovered, with restoration of normal hearing. The third patient of this group was extremely nervous and trembling. Prostration was early quite marked, and a lung complication carried him off on the fourteenth day in hospital. Our management of typhoid cases (none but well-defined cases have been so designated) has been to keep a careful register of the temperature, pulse and respiration; and to diminish the high temperature by frequent bathing or sponging; moderate the diarrhœa, if any existed, by turpentine emulsion and tinct. opii, and by external applications over the bowels, of lard and turpentine with moist heat in cases of much tenderness; keep up the action of the kidneys with a mild alkaline diuretic; and give a supporting diet, of milk principally, frequently administered. Stimulants were not given at all in the earlier stages of the disease, and seldom in the latter. In cases of pulmonary complications the treatment was adapted to the general indications—turpentine applications, covered with flannel wrung out of hot water. One case had, near the close of the disease, suppuration of the left parotid gland. Patient was a young man of delicate constitution, light complexion and red hair. This is the third case of the kind I have had in my own practice, all of whom, however, recovered, notwithstanding it is regarded as a rare complication. See Trousseau, also Da Costa.

The case of tuberculosis, complicated with hydrothorax, was a colored boy twenty years of age. He had had a severe and prolonged attack of pheumonia about one year before, but had recovered sufficiently to do work most of the summer. He entered hospital December 23. By January 1, respiration became exceeding difficult. With the aspirator I drew off twenty-three ounces of fluid from the right pleural cavity, which afforded him great relief, though of temporary duration. On the 4th of February the pressure from accumulation of fluid became so great as to demand relief, and I drew off fifty-five ounces. The operation was repeated, at intervals of from five to eight days, until March 1. The fluid drawn was bloody serum of 1018 to 1022 sp. gravity. Under the microscope the blood-corpuscles appeared crenated, and evidently many of them disintegrating. The entire quantity drawn was two hundred and forty ounces. The pulse ranged from 90 to 120, except occasionally and during the last few days, when it ran higher.

The post-mortem revealed the right pleural cavity filled with reddish serum; the lung was contracted to the size of a fist, and containing many tubercular masses; some adhesions were stretching across the chest wall. The left cavity also contained considerable serum, as did also the abdominal cavity; and the lung was filled with tubercles. It is easy to see how, by means of these adhesions, exploratory tapping with the hypodermic syringe might have failed to reach the fluid in the thoracic cavity. Tuberculosis, rapid in its course, was the incurable principle of his illness; and hydrothorax the incidental, for which thoracentesis gave great but only temporary relief.

The other case of acute tuberculosis (Frank Kennedy) was very rapid in its course. One marked feature of the early history of the case was a wide deviation from the normal pulse-respiration ratio. On being transferred to the hospital from the sick-cell, I found his pulse varying from 100 to 110; and yet the respiration was from 40 to 50, and even at some times as high as 60. Expectoration was very slight. Treatment seemed to be of no benefit.

Post-mortem revealed both lungs literally crowded with tubercles, from the size of a pin head to half the size of a pea. This condition of the lung explains the clinical symptom of rapid respiration, and suffering from a "want of breath," which was at times very distressing.

Charles Moseley, colored, died July 21, 1882, at 11:50 A. M. Autopsy three and a half hours after death. Body well nourished. Rigor mortis scarcely perceptible: large scar on right wrist; large perpendicular scar on the chest, four inches long, above the left nipple. Blister eight inches in diameter over the region of the stomach.

An incision was carried from the upper border of the sternum to the symphysis pubis. The blood which escaped from the incised vessels was extremely watery. Ecchymosis existed on the under side of the rectus muscles of the abdomen, about two and a half inches in diameter, and situated two inches above the umbilicus. The lungs, collapsed, were free, and contained very little blood, except the base of the left, which was very dark, and contained considerable very dark blood; but a piece of it floated in water. It seems to be the result of hypostasis. The left side of the heart was contracted, but was entirely empty; the right side was flabby (did not open it). The liver was large (did not weigh it), and filled with very dark, thick blood. On section it was of a lighter yellow than normal, in parts quite decided well marked in this respect—fatty degeneration. The pancreas was very large, but did not seem to be otherwise abnormal, except rather dense. Weight, 6½ ounces. The kidneys were large, and the right had ecchymosed spots on and in the cortical portion. It was of a yellowish white color, and bore the appearance of acute fatty degeneration. The left bore the same marks, but in less degree. The bladder was partially filled with urine, which, on examination, proved to contain albumen ($\frac{12}{20}$). The stomach was much contracted, and contained a dark greenish fluid; the mucous membrane was much tumefied, but no ulceration was perceptible.

I have given the *post-mortem* before the clinical history because on the former we finally based the diagnosis.

Clinical history: On the night of the 6th and 7th of July (about midnight) he was visited; he had been vomiting freely, as he said and as the condition of his cell indicated. A prominent and persistent symptom was nausea and frequent vomiting—at intervals of a half hour to four or five hours. Nothing seemed capable of allaying it for any length of time. The ejected matter was of a dark-green color, mixed with whatever he may have taken to eat—milk once or twice. He spat blood at times. It would seem to accumulate in the back part of the mouth, and he would spit it out without any effort at vomiting. About the time he came into hospital, for a day or two, it would come pretty freely with slight effort at vomiting; but, in smaller quantities, it would be as likely to come after vomiting. Another symptom of unusual character, in relation to other conditions, was the absence of fever during the entire course of the attack, except a very few hours before death.

Also, we may mention the slow, regular pulse, not exceeding 70 during the entire time except during the last few hours. I was fully persuaded that it was a case of poisoning of some kind, but the only poisons occurring to me as accessible to him were corrosive sublimate, concentrated lye and fish-berries (*anamirta cocculus*). The symptoms, however, did not correspond to any of them, and the delay in manifestation obscured the case. Phosphorus did not occur to me until the *post mortem*. The convicts are not allowed matches; but he being in the female department, to help iron and make fires, had access to matches constantly. It will be seen that nearly all the *post-mortem* appearances were those of phosphorus poisoning. The duration of the case is rather unusual, though others are on record of even greater duration, and yet fatal. His previous health had been perfect, and he was in every way stout. He was always of a reticent character; and during his illness it was with the greatest reluctance that he would say anything about his case. He was serving a second term.

Summary of Monthly Reports of the Medical Department of the Southern Illinois Penitentiary, Chester, Illinois, for the year commencing August 1, 1881, and ending July 31, 1882. Services of H. Z. GILL, M. D., Physician to Southern Illinois Penitentiary.

The number of convicts increased, during the year from August, 1881, to July, 1882, inclusive, from an average of 356.5 to 513, giving an average for the year of 436. The health was generally good. The rate of mortality per annum for 1,000 men, was 27.5; and deducting the case of poisoning (suicide), 25.23. New York gave, 1881, 31.7.

The loss of time last summer was much greater than during this summer, it being then much hotter. Lost time, July, 1881, 710.75; number convicts, 354; per cent., 7.72. Lost time, July, 1882, 346.75; number convicts, 513; per cent., 2.6.

The cases, Nos. 8, 9, 10, 11, are the only ones that can be properly classed as among diseases measurably under the influence of treatment. No. 1 was thoroughly poisoned, and his health broken down by long confinement in jail before coming to this institution. See below farther description of the individual cases.

[SEE NEXT PAGE FOR TABLES.]

### SUMMARY OF MONTHLY REPORTS.

| MONTHS | AUGUST. | SEPT'R. | OCT'R. | NOV'R. |
|---|---|---|---|---|
| Average number convicts | 356.5 | 354 | 347.7 | 357.7 |
| Mean monthly temperature (this is approx. correct) | 83 | 76.7 | 62 5 | 43 |
| Average number attending daily sick call | 38 | 30.6 | 17.8 | 14.6 |
| Average number in hospital | 10.8 | 10 | 7.1 | 6.3 |
| Average number in sick cell | 4.4 | 3 | 2.6 | 2 |
| Working days lost from sickness— | | | | |
| 1. In hospital | 293 | 261 | 184.5 | 165 |
| 2. In sick cell | 128.5 | 76.6 | 70.3 | 54 |
| Total lost time | 421.5 | 337.6 | 254.8 | 219 |
| Deaths | 1 | 1 | | |
| Sent to insane asylum | 1 | | | |
| Antiperiodics used | $25.60 | $18.00 | $12.40 | $12.40 |
| Number men fed by medical department— | | | | |
| 1. In hospital | | 307.5 | 317 | 253 |
| 2. In cell house | | 107.5 | 112 | 122 |
| Total | | 415 | 429 | 375 |
| Gallons of milk issued to sick | | 55 | 90 | 85 |
| DISEASES—1. MEDICAL. | | | | |
| Intermittent fever | 17 | 4 | 3 | 1 |
| Remittent fever | | 2 | 5 | 3 |
| Continued fever | | | | 2 |
| Malarial fever | 1 | | | |
| Typho-malarial fever | | | 1 | 1 |
| Typhoid fever | | | | |
| Diphtheria | | | | |
| Pneumonia | | | | |
| Dirarhœa, acute | 2 | 2 | | 2 |
| " chronic | 1 | | 1 | |
| Bright's disease, acute | | | | |
| " chronic | 1 | | | |
| Rheumatism | 4 | 3 | | 2 |
| Epilepsy | | | | |
| Consumption | | | | 1 |
| Jaundice | | | | |
| 2. SURGICAL. | | | | |
| Hæmorrhoids | | 3 | 2 | 3 |
| Fistula in ano | | 1 | 1 | |
| Sprains | 1 | | | |
| Fractures | | | | |
| Injuries and contusions | 1 | 3 | | 1 |
| Floating cartilage in knee-joint | | | | |
| SURGICAL OPERATIONS. | | | | |
| Removal of right eye | | 1 | | |
| " tumor from face | | | 1 | |
| Hæmorrhoids | | 3 | 2 | 3 |
| Fistula in ano | | 1 | 1 | |
| " lachrymal | | | | |
| Paracentesis, abdominal | | 1 | 2 | |
| " thoracis | | | | |
| Operations on eye-lids | | 1 | | |
| Palmar abscess | | | | |
| Fracture in elbow | | | | |
| " of right leg | | | | |
| " of clavicle | | | | |
| Strangulated hernia, inguinal | | | | |
| Bubo (syphilitic) | | | | |
| Removal of bullets | | | | |
| " of floating cartilage from knee-joint | | | | |
| Amputation of toe | | | | |

SUMMARY OF MONTHLY REPORTS—*Concluded.*

| DEC'R. | JAN'Y. | FEB'Y. | MARCH. | APRIL. | MAY. | JUNE. | JULY. | Total or Av'ge. |
|---|---|---|---|---|---|---|---|---|
| 382 | 389 | 383.7 | 419 | 449.6 | 476.3 | 504.2 | 513 | 436 |
| 40 | 37.6 | 49.1 | 48.2 | 59.3 | 65.8 | 79.5 | 79.7 | ........ |
| 16.3 | 27 | 32.5 | 24.6 | 30 | 25.5 | 23 | 20.5 | ........ |
| 9.3 | 10.2 | 6.5 | 6.9 | 7.8 | 8.2 | 8.2 | 8.1 | ........ |
| 3.5 | 5.3 | 3.5 | 2.1 | 6 | 7.6 | 4.3 | 5.2 | ........ |
| 251 | 262 | 151.5 | 186 | 195 | 220 | 214 | 211 | ........ |
| 94.6 | 136.6 | 83.5 | 59 | 149 | 206.5 | 110.6 | 135.6 | ........ |
| 354.6 | 398.6 | 235 | 245 | 344 | 426.5 | 324.6 | 346.6 | ........ |
| 1 | 1 | ........ | 3 | 1 | 1 | 1 | 2 | 12 |
| ........ | ........ | 1 | ........ | ........ | ........ | ........ | ........ | ........ |
| $12.40 | $9.70 | $13.10 | $15.50 | $7.50 | $11.80 | $6.00 | $7.45 | ........ |
| 444 | 444 | 292 | 338 | 353 | 356 | 311 | 306 | ........ |
| 241 | 253 | 288 | 297 | 346 | 381 | 396 | 382 | ........ |
| 685 | 697 | 580 | 635 | 699 | 7 7 | 707 | 688 | ........ |
| 101 | 112 | 112 | 123 | 128 | 98.5 | ........ | ........ | ........ |
| 2 | 2 | 3 | 10 | 8 | 4 | 4 | 4 | ........ |
| 3 | 2 | 5 | 3 | 4 | 3 | 1 | ........ | ........ |
| ........ | ........ | ........ | ........ | 2 | 1 | 2 | ........ | ........ |
| ........ | ........ | 1 | ........ | ........ | 1 | ........ | ........ | ........ |
| ........ | 2 | ........ | ........ | ........ | ........ | ........ | ........ | ........ |
| 2 | 2 | ........ | ........ | 3 | ........ | ........ | 1 | 8 |
| ........ | ........ | ........ | ........ | ........ | ........ | 1 | ........ | ........ |
| ........ | ........ | ........ | 1 | ........ | 2 | 1 | ........ | ........ |
| 1 | 1 | ........ | 3 | ........ | 1 | 1 | 2 | ........ |
| 1 | ........ | ........ | 5 | 2 | ........ | 1 | 3 | ........ |
| 1 | ........ | ........ | 1 | ........ | ........ | ........ | ........ | ........ |
| ........ | ........ | ........ | ........ | ........ | ........ | ........ | ........ | ........ |
| 3 | ........ | 1 | ........ | 1 | 26 | 5 | 1 | ........ |
| ........ | 2 | ........ | ........ | ........ | 1 | ........ | ........ | ........ |
| 1 | ........ | 1 | ........ | ........ | ........ | ........ | ........ | ........ |
| ........ | ........ | 1 | ........ | 1 | ........ | ........ | 1 | ........ |
| 6 | 3 | 1 | ........ | ........ | ........ | ........ | 1 | ........ |
| ........ | ........ | ........ | ........ | ........ | ........ | 1 | ........ | ........ |
| ........ | ........ | ........ | ........ | ........ | ........ | 1 | 1 | ........ |
| 1 | 1 | ........ | 1 | ........ | ........ | ........ | ........ | ........ |
| ........ | 1 | ........ | 1 | ........ | ........ | 1 | ........ | ........ |
| ........ | ........ | ........ | 1 | ........ | ........ | ........ | ........ | ........ |
| ........ | ........ | ........ | ........ | ........ | ........ | ........ | ........ | ........ |
| ........ | ........ | ........ | ........ | ........ | ........ | ........ | ........ | ........ |
| 6 | 3 | 1 | ........ | ........ | ........ | ........ | 1 | 20 |
| ........ | ........ | ........ | ........ | ........ | ........ | 1 | ........ | ........ |
| 1 | ........ | ........ | ........ | ........ | ........ | ........ | ........ | ........ |
| ........ | ........ | ........ | ........ | ........ | ........ | ........ | ........ | ........ |
| ........ | 1 | 4 | 1 | ........ | ........ | ........ | ........ | ........ |
| ........ | ........ | ........ | 1 | ........ | ........ | ........ | ........ | ........ |
| ........ | 1 | ........ | ........ | ........ | ........ | ........ | ........ | ........ |
| 1 | ........ | ........ | ........ | ........ | ........ | ........ | ........ | ........ |
| ........ | 1 | ........ | 1 | ........ | ........ | ........ | ........ | ........ |
| ........ | 1 | ........ | 1 | 1 | ........ | ........ | ........ | ........ |
| ........ | 1 | ........ | 1 | ........ | ........ | ........ | ........ | ........ |
| 1 | ........ | ........ | ........ | ........ | ........ | ........ | ........ | ........ |
| ........ | 1 | ........ | ........ | ........ | 1 | ........ | ........ | ........ |
| ........ | ........ | ........ | 1 | ........ | ........ | ........ | ........ | ........ |
| ........ | ........ | ........ | ........ | ........ | ........ | ........ | 1 | ........ |

